# Arp2/3 complex activity in filopodia of spreading cells

**DOI:** 10.1186/1471-2121-9-65

**Published:** 2008-12-09

**Authors:** Simon A Johnston, Jonathan P Bramble, Chun L Yeung, Paula M Mendes, Laura M Machesky

**Affiliations:** 1 University of Birmingham School of Biosciences, Edgbaston, Birmingham, B15 2TT, UK; 2 Department of Chemical Engineering, Edgbaston, Birmingham, B15 2TT, UK; 3 CRUK Beatson Institute for Cancer Research, Garscube Estate, Switchback Rd, Bearsden, Glasgow, G63 9AE, UK; 4 Molecular and Nanoscale Physics Group, School of Physics and Astronomy, University of Leeds, Woodhouse Lane, Leeds, LS2 9JT, UK

## Abstract

**Background:**

Cells use filopodia to explore their environment and to form new adhesion contacts for motility and spreading. The Arp2/3 complex has been implicated in lamellipodial actin assembly as a major nucleator of new actin filaments in branched networks. The interplay between filopodial and lamellipodial protrusions is an area of much interest as it is thought to be a key determinant of how cells make motility choices.

**Results:**

We find that Arp2/3 complex localises to dynamic puncta in filopodia as well as lamellipodia of spreading cells. Arp2/3 complex spots do not appear to depend on local adhesion or on microtubules for their localisation but their inclusion in filopodia or lamellipodia depends on the activity of the small GTPase Rac1. Arp2/3 complex spots in filopodia are capable of incorporating monomeric actin, suggesting the presence of available filament barbed ends for polymerisation. Arp2/3 complex in filopodia co-localises with lamellipodial proteins such as capping protein and cortactin. The dynamics of Arp2/3 complex puncta suggests that they are moving bi-directionally along the length of filopodia and that they may be regions of lamellipodial activity within the filopodia.

**Conclusion:**

We suggest that filopodia of spreading cells have regions of lamellipodial activity and that this activity affects the morphology and movement of filopodia. Our work has implications for how we understand the interplay between lamellipodia and filopodia and for how actin networks are generated spatially in cells.

## Background

Two major types of structures dominate actin formations in cells: unbranched filaments that are bundled together and branched networks of unbundled filaments. In the migrating cell, the leading edge contains both types of actin structure, branched networks of actin filaments that form lamellipodia and parallel bundles that form filopodia.

The Arp2/3 complex, a seven subunit complex containing two actin-related proteins (Arp2 and Arp3), is responsible for the generation of branched networks of actin filaments. Arp2/3 complex cooperates with proteins such as cofilin and gelsolin that make available actin templates for Arp2/3 complex to form branches (Reviewed in [1]). The Arp2/3 complex is activated by WASP-family proteins via a variety of different signal inputs such as activation of tyrosine kinase receptors and small GTPases of the Rho-family (Reviewed in [2]). Activation of Arp2/3 complex increases its binding to the sides of actin filaments and induces the formation of an actin branch, which grows and is thought to push against the plasma membrane causing lamellipodial protrusions. Lamellipodial networks are subsequently reorganised so that the cell can form specialised structures for motility.

It has been proposed that filopodia are generated by the reorganisation of an Arp2/3 complex generated actin network [3,4]. Proteins that mediate filament elongation (e.g. VASP and formins) bind to actin filaments in lamellipodia and come together to form a filopodial tip complex that elongates a group of actin filaments forming a filopodium. However, lamellipodia do not seem to be essential for filopodia formation, as filopodia form regardless of loss of most of the Arp2/3 complex or the Scar/WAVE complex in mammalian cells or in *Dictyostelium *[5]. Furthermore, it seems unlikely that all filopodia are largely dependent on a dendritic network, as spreading fibroblasts and platelets clearly produce filopodia in the absence of detectable lamellipodia [6,7].

Here, we describe the localisation of the Arp2/3 complex to filopodia in a manner that is apparently independent of adhesion but coincident with the localisation of other lamellipodial proteins to filopodia and dependent on Rac1 signaling. We suggest that filopodia in spreading cells may contain regions of lamellipodial activity, which could offer the cell flexibility in its motility choices.

## Results

### The Arp2/3 complex localises to filopodia during cell spreading

After attachment to the fibronectin substrate, mouse embryonic fibroblasts (MEFs) produced filopodia within 5 minutes (see Additional file 1). Filopodia production is followed by extension of lamellipodia and cell spreading continued with repeated cycles of filopodia and lamellipodia assembly. Once lamellipodial extension had begun, the spread area of the cell continually increased until 30 minutes after plating, with very little or no retraction of the cell perimeter (see Additional file 1 and 2). Between 30 and 60 minutes MEFs began to form a more characteristic fibroblast shape but there was little change in the total cell area (see Additional file 2).

**Figure 1:**
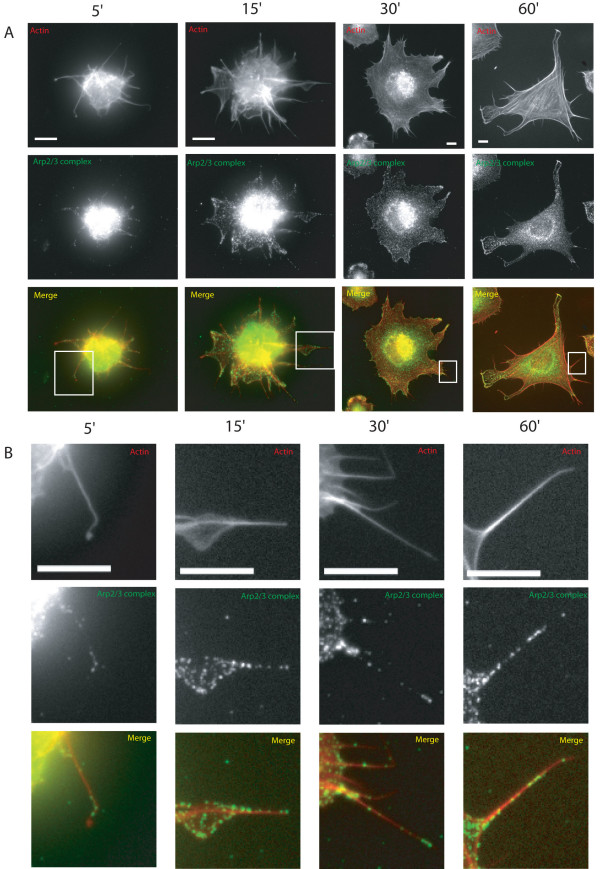
**The localisation of the Arp2/3 complex in spreading mouse embryonic fibrobloasts**. MEFs were plated onto fibronectin and fixed at 5, 15, 30 and 60 minutes. Actin was labelled with rhodamine phalloidin and Arp2/3 complex with an antibody to the p34-Arc subunit. Boxed areas are enlarged in B. Scale bar 10 μm.

We aimed to determine the involvement of Arp2/3 complex in cell spreading, so first we localised Arp2/3 complex in spreading MEF. Figure 1A shows a sequence of representative photos of Arp2/3 complex localization during a spreading time course. At approximately 5 minutes after plating, before lamellipodia were formed, Arp2/3 complex had a punctate localisation in the cytoplasm. From 15 minutes onwards Arp2/3 complex was at the edge of the cell as well as cytoplasmic. Between 15 and 30 minutes, the localisation of Arp2/3 complex to the cell cortex became more distinct and lamellipodia were present at multiple sites. By 60 minutes, Arp2/3 complex was usually restricted to a few well-defined lamellipodial protrusions with intense labelling.

Unexpectedly, Arp2/3 complex was also localised to some of the filopodia-like actin protrusions (Figure 1B, enlargements of boxed areas) produced during spreading. A monoclonal antibody to Arp2 showed near identical labelling to the p34-arc antibody in lamellipodia, on cytoplasmic spots and in filopodia (see Additional file 3). We identified the protrusions as filopodia by expressing the filopodial actin bundling protein fascin [8,9] tagged with GFP in spreading cells. In cells expressing relatively low levels of fascin, cell morphology was not affected (data not shown). In low expressing cells, fascin labelled the filopodial-like protrusions that contained Arp2/3 complex but fascin and Arp2/3 complex were not especially coincident in filopodia (data not shown).

**Figure 2:**
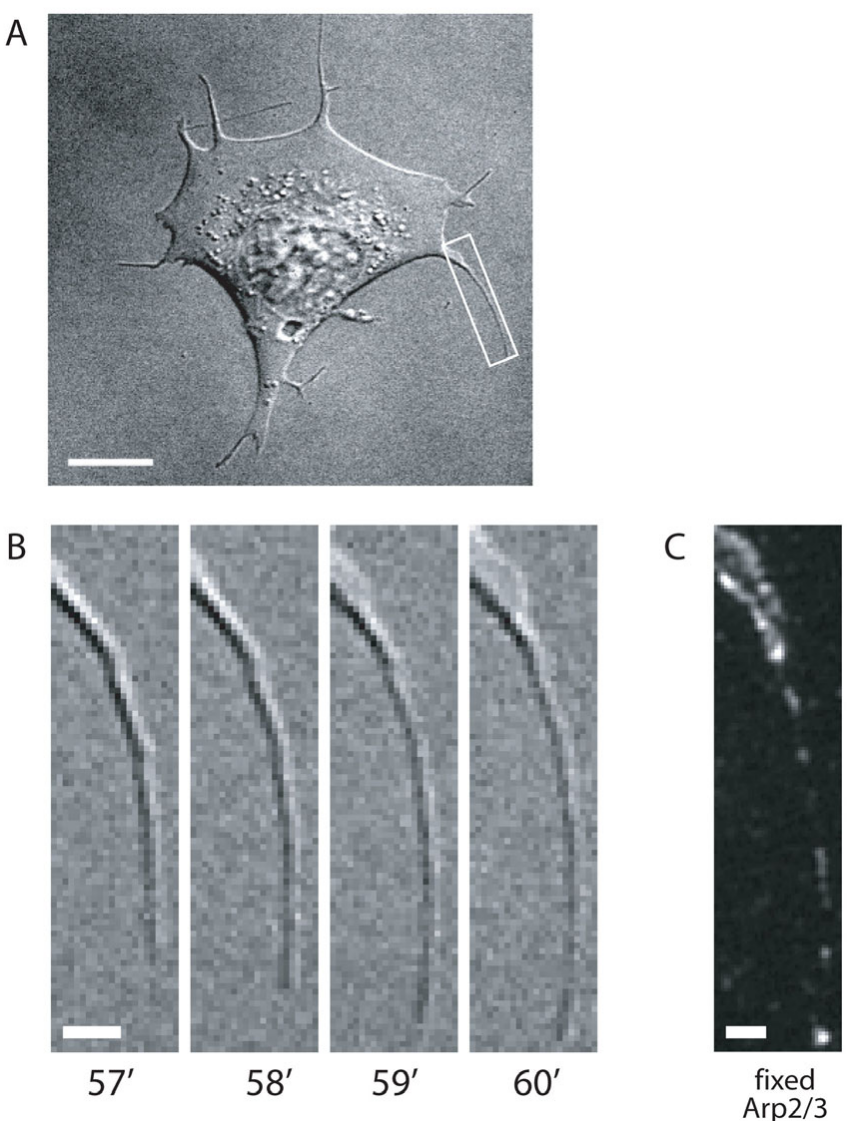
**Arp2/3 complex localises to actively protruding filopodia**. A. DIC image taken 60' after plating and prior to fixation. Box indicates area shown in B. Scale bar 10 μm. B. Frames taken from DIC timelapse at times indicated after plating. Scale bar 2 μm. C. Arp2/3 complex labelling in the same filopodium pictured in B and shown in Movie 2. Scale bar 2 μm.

We next examined the dynamics of the actin structures that contained Arp2/3 complex to confirm that they behaved as typical filopodia- i.e. thin projections that grew from the cell edge and whose tips moved relative to the cell body [10]. Arp2/3 complex containing filopodia were identified by immunofluorescence and these same filopodia found in correlative DIC time lapses. At least some of the filopodia labelled with Arp2/3 complex were actively protruding filopodia (Figure 2, see Additional file 4). The behaviour of the structures labelled with Arp2/3 complex, together with the molecular evidence from the labelling with fascin, confirmed that we were frequently observing genuine filopodia that contained multiple spots of Arp2/3 complex.

**Figure 3:**
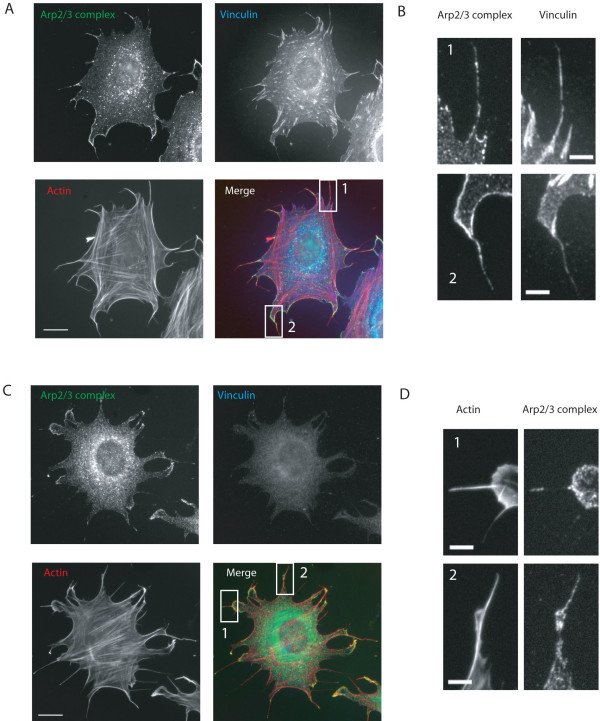
**Arp2/3 complex and vinculin in filopodia**. A. MEF spread on fibronectin for 60 minutes. Labelling for vinculin showed numerous focal adhesions. Scale bar 20 μm. B. Enlargements of boxed areas in A. Vinculin labelling of filopodia is variable in intensity and not comparable to Arp2/3 complex labelling. Scale bar 5 μm. C. vinculin null MEF spread on fibronectin for 60 minutes. Labelling with vinculin antibody shows complete loss of vinculin labelling. Actin was labelled with rhodamine phalloidin and Arp2/3 complex with an antibody to the p34-Arc subunit. Scale bar 20 μm. D. Enlargements of boxed areas in A. Scale bar 5 μm.

Having established these structures as filopodia, we measured their occurrence during cell spreading. At 15 and 30 minutes about 30% of filopodia contained Arp2/3 complex and by 60 minutes this had increased to 40% (Table 1). Correlative DIC time-lapse and immunofluorescence microscopy showed that discounting retraction fibres at 60 minutes, cells had 36% Arp2/3 complex containing filopodia, which agrees with data from fixed cell studies (Table 1). Arp2/3 complex is present in filopodia at the earliest stages of spreading and, as with lamellipodia, filopodia were more frequent and strongly labelled by Arp2/3 complex at 30 and 60 minutes (Figure 3A, 60 minutes time point). A range of cell types contained Arp2/3 complex in their filopodia, including J774 macrophages, HeLa, Human embryonic lung fibroblasts (data not shown) and the social amoeba *Dictyostelium *[11]. Thus, the localisation of Arp2/3 complex to filopodia seems to be a general but often overlooked feature of cytoskeletal organization.

**Table 1:** Average number of filopodia and filopodia containing Arp2/3 complex per cell.

**Average number per cell**	**15 minutes**	**30 minutes**	**60 minutes**
Filopodia	11.7 (± 2.5)	14.1 (± 1.3)	17.3 (± 3.1)
Filopodia containing Arp2/3 complex	3.2 (± 1.4)	3.9 (± 1.4)	7.0 (± 0.4)

*Percentage*	*27.4*	*26.2*	*40.5*

Cells were spread on fibronectin for the ties indicated before fixation. Counts are from 3 independent experiments and are ± standard deviation. 15 minutes n = 73, 30 minutes n = 50, 60 minutes n = 30.

### The dynamics of Arp2/3 complex in filopodia suggested regions of lamellipodial activity within filopodia

**Figure 4:**
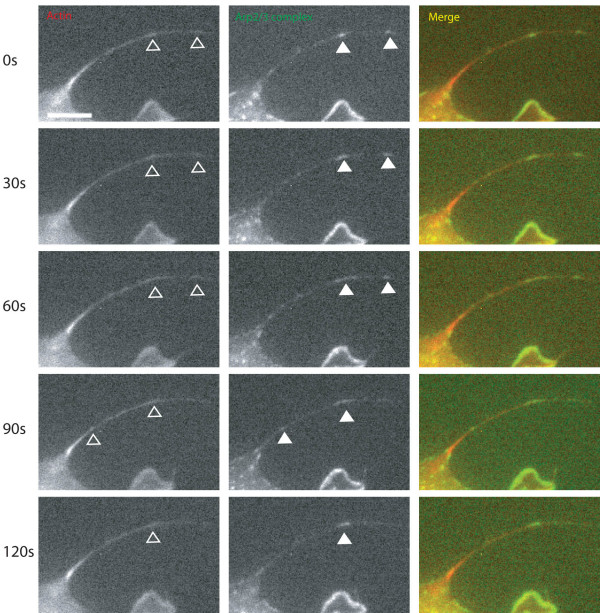
**Arp2/3 complex patches in filopodia are highly dynamic and co-localise with increases in RFP actin signal**. Cells were spread for 60 minutes on fibronectin after co-transfection with GFP p21-arc and RFP actin. Solid arrows indicate Arp2/3 complex foci in filopodia and hollow arrows indicate the corresponding location in the RFP actin images. Images are taken from Movie 4. Scale bar 10 μm.

Lamellipodia are protrusions of membrane driven by dendritic networks of actin, while filopodia are classically thought to be driven by parallel bundles of elongating actin filaments. We examined the dynamics of Arp2/3 complex in filopodia during cell spreading using p21-arc subunit of the Arp2/3 complex tagged with GFP. RFP actin was co-expressed to confirm the location of filopodia. The patches of Arp2/3 complex in filopodia were highly dynamic, changing size and intensity much in the same way as lamellipodial ruffles (Figure 4 and see Additional file 5 and 6). Arp2/3 complex puncta appeared and disappeared within filopodia, rather than seeming to be transported from within the cell or from lamellipodia.

From time-lapse fluorescent imaging, Arp2/3 complex was present in both filopodia extending from a dynamic leading edge (see Additional file 7A) and in less dynamic filopodia present at previously retracted cell edges (see Additional file 7B). Of the several filopodia containing Arp2/3 complex analyzed (53 Arp2/3 complex containing filopodia, 11 cells) by live imaging the patches did not apparently become larger areas of protrusion even when positioned immediately in front of the leading edge. Therefore, we do not think that individual small Arp2/3 complex patches that we have observed in filopodia lead to a larger lamellipodial expansion, but we cannot exclude the possibility that this is a rare event or that coalescence of several patches might not lead to lamellipodial expansion.

### Arp2/3 complex localisation to filopodia is not dependent on vinculin, microtubules or local interaction with a fibronectin surface

The focal adhesion protein vinculin has been reported to directly associate with the Arp2/3 complex during spreading [12] and therefore we looked at the localisation of vinculin and Arp2/3 complex in filopodia. Filopodia were labelled with vinculin in varying degrees in some cases as intensely as in focal adhesions (Figure 3A). Arp2/3 complex and vinculin were occasionally coincident in filopodia but did not show the same distribution (Figure 3B). We observed this general staining pattern in over 200 cells in three separate experiments. We concluded that vinculin did not strongly co-localise with Arp2/3 complex in filopodia during the first 60 minutes of spreading. To further explore the potential involvement of vinculin in localizing Arp2/3 complex to filopodia we made use of vinculin null MEFs. After 60 minutes cells lacking vinculin had similar morphology to wild type cells (Figure 3C; [13,14]) with 33% of filopodia containing Arp2/3 complex at 60 minutes also comparable to wild type cells (Figure 3D; data not shown). Paxillin, also part of the focal adhesion complex, similarly showed some coincidence with Arp2/3 complex but no clear co-localisation (data not shown). Thus, neither vinculin nor paxillin were reliably co-localised with Arp2/3 complex and vinculin was not required for localising Arp2/3 complex to filopodia. Disruption of microtubules also did not have any effect on the localization of Arp2/3 complex in filopodia, although the morphology of the filopodia was altered, with cells displaying many clubbed or bent filopodia (data not shown).

By using BSA to block cell adhesion after initial attachment to a glass substrate [15] we could assess the cell adhesion requirements of Arp2/3 complex localisation. After 60 minutes, cells did not have lamellipodia and no localisation of Arp2/3 complex to the cortex was seen (see Additional file 8). Filopodia were abundant and the majority of them were clubbed (see Additional file 8) as with inhibition of microtubules (data not shown). This suggested that lamellipodia but not filopodia were dependent on an adhesive surface.

To further explore the role of adhesion in Arp2/3 complex recruitment to filopodia, we designed a micropatterned surface that contained both adhesive and non-adhesive areas. This had the advantage of activating outside-in signaling pathways while enabling us to examine how the cell interacted with non-adhesive areas. To visualise the pattern, fibronectin was labelled with the fluorophore Cy3 and we confirmed that MEFs showed normal spreading, Arp2/3 complex localisation and adhesion formation on a homogenous Cy3 fibronectin surface (data not shown). MEF cells were able to spread on both 5 and 10 μm stripes and the orientation of spreading was always in the direction of the striped pattern (see Additional file 9)

**Figure 5:**
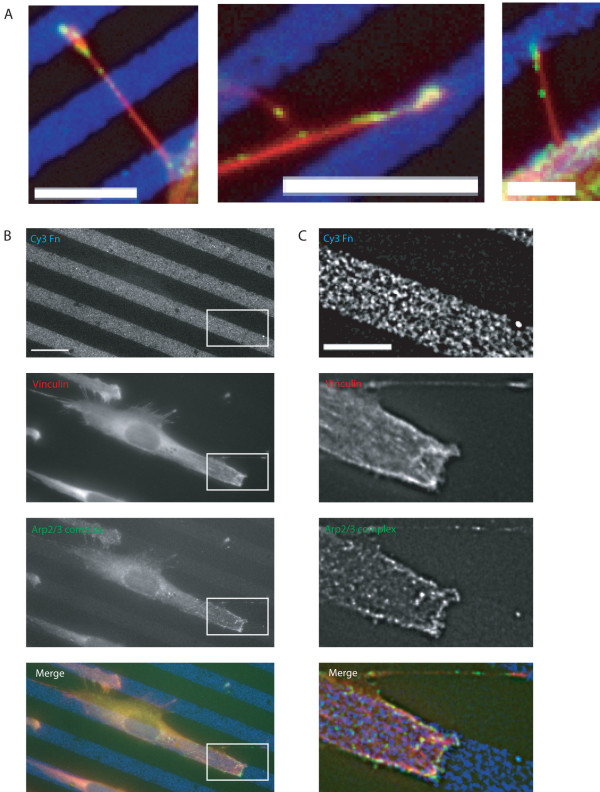
**Arp2/3 complex localisation to filopodia is independent of adhesion**. A. Filopodia of MEF spread on 5 μm fibronectin stripes for 60 minutes. Arp2/3 complex is present on BSA stripes. Blue, Cy3 fibronectin, green, Arp2/3 complex (antibody to p34-Arc sub-unit) and red (rhodamine phalloidin), actin. Left and middle panel scale bar 10 μm, right panel 5 μm. B. MEF spread on 10 μm fibronectin stripes for 60 minutes. Scale bar 10 μm. C. Enlargement and deconvolution of boxed area in B. Scale bar 10 μm.

Cells spread on these patterned surfaces frequently protruded filopodia into non-adhesive areas (see Additional file 9). Vinculin was absent from areas of filopodia that were extended over non-adhesive areas, whereas Arp2/3 complex was often present (Figure 5). We concluded that the localisation of Arp2/3 complex to filopodia is independent of local adhesion to a fibronectin surface.

### Arp2/3 complex in filopodia is present with cortactin and at the sites of free barbed ends

**Figure 6:**
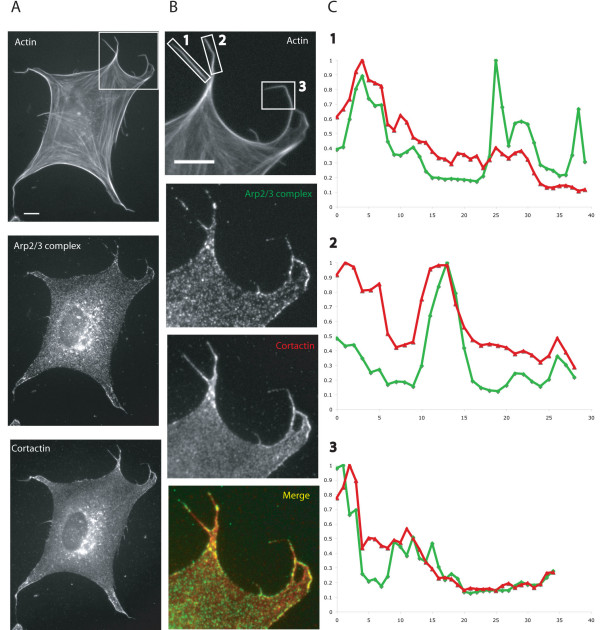
**Cortactin labels the protruding edges of spreading MEFs and is coincident with Arp2/3 complex**. A. MEFs were spread for 60 minutes on fibronectin. Scale bar 10 μm. B. Enlargement of boxed area in A. Merge is of cortactin (4F11 antibody) and Arp2/3 complex (antibody to p34-Arc sub-unit). Scale bar 10 μm. C. Intensity of cortactin (red line) and Arp2/3 complex (green line) signals relative to respective maximum (set at 1) values along the lengths (arbitrary units) of boxed filopodia in B.

Cortactin functions with Arp2/3 complex to form branched actin networks and therefore when present at the cell cortex is a lamellipodial marker [4]. Anti-cortactin strongly labelled both lamellipodia and filopodia (Figure 6) and increases in cortactin labelling were often coincident with similar increases in Arp2/3 complex labelling in distinct areas (Figure 6C).

**Figure 7:**
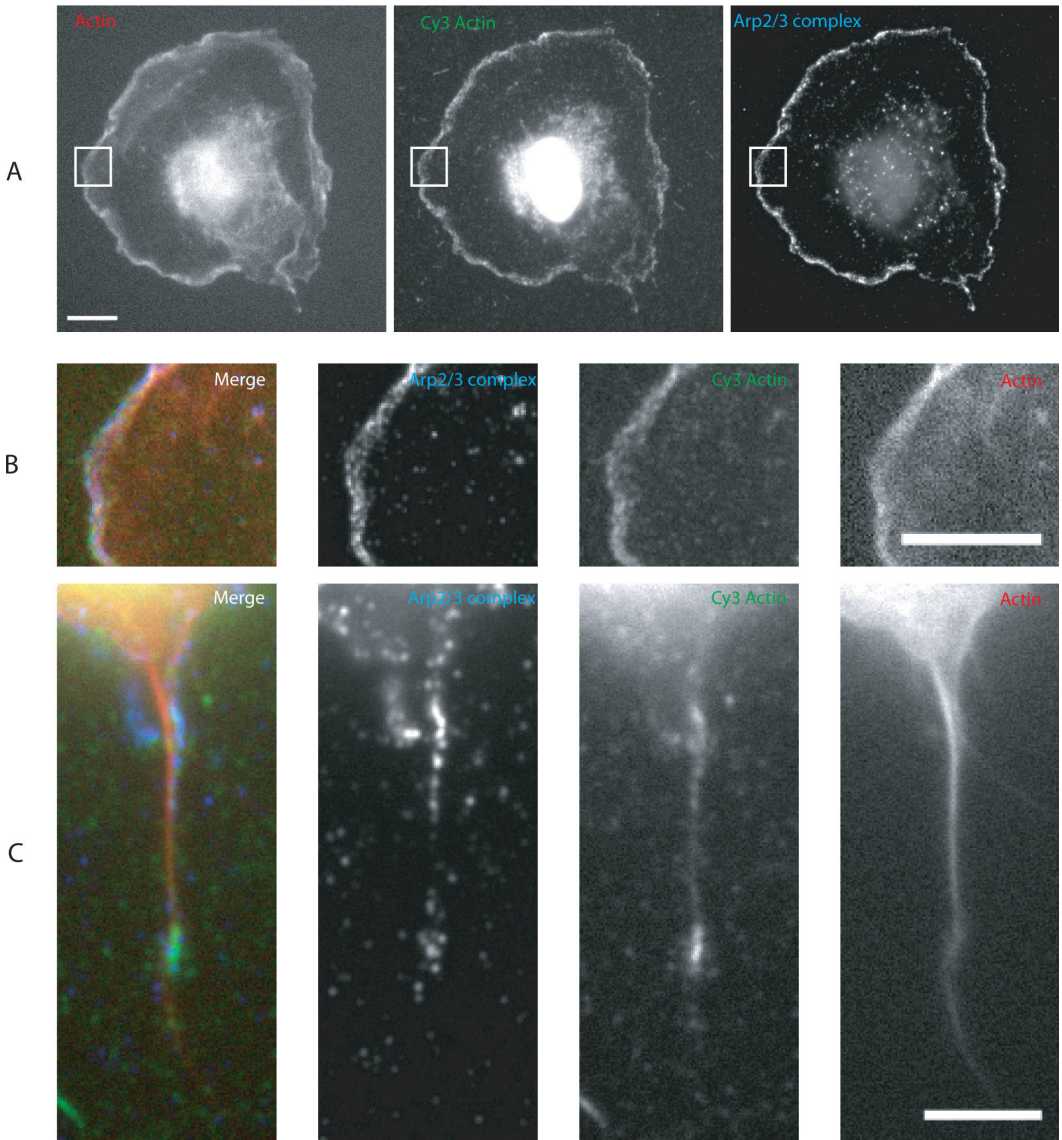
**Cy3 labelled actin monomers incorporate into sites of Arp2/3 complex localisation in filopodia**. Cells were spread for 60 minutes on fibronectin, permiabilised in the presence of Cy3 labelled actin and fixed. A. Incorporation of Cy3 actin to the cortex and nucleus of MEF spread for 60 minutes on fibronectin. Nuclear labelling has been reported previously with this assay [16] Scale bar 10 μm. B. Enlargement of boxed area in A. showing Cy3 actin incorporation into lamellipodia and cytoplasmic actin spots. Scale bar 5 μm. C. Cy3 actin incorporation into sites of Arp2/3 complex localisation in filopodia. Actin was labelled with rhodamine phalloidin and Arp2/3 complex with an antibody to the p34-Arc subunit. Scale bar 5 μm.

The presence of cortactin suggested that Arp2/3 complex was active and generating an actin network. Actin monomers fluorescently labelled with Cy3 were used to probe for free barbed ends at these sites [16]. Labelled actin was incorporated into lamellipodia of spreading cells (Figure 7A, B) and also into sites of Arp2/3 complex localisation in filopodia (Figure 7C). Other sites in filopodia were not labelled showing that free barbed ends are not concentrated elsewhere in filopodia.

### The localisation of Arp2/3 complex to filopodia is regulated by Rac activity

**Figure 8:**
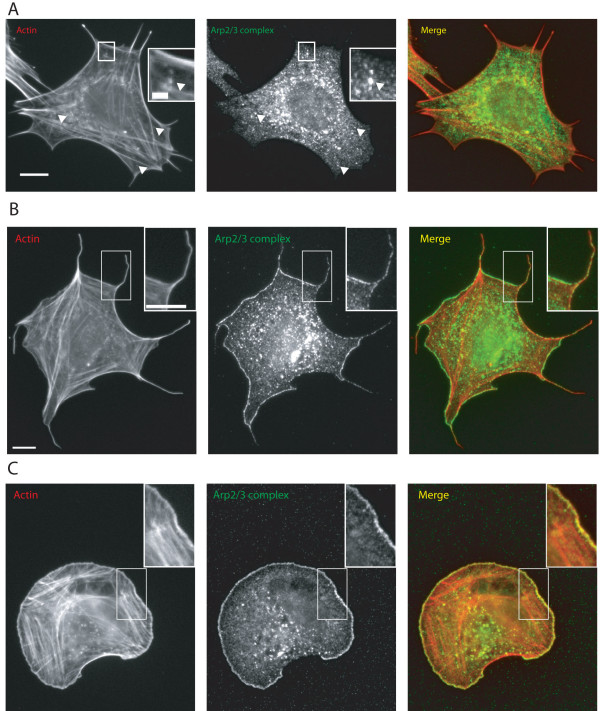
**A. Effect of dominant negative mutant of Rac1 T17N on localisation of Arp2/3 complex in cell spreading**. MEFs were transfected with myc tagged T17N Rac 1 and spread for 60 minutes on fibronectin. Scale bar 10 μm. Inset 2 μm B, C. Effect of constitutively active mutant of Rac 1 Q61L on localisation of Arp2/3 complex in cell spreading. Scale bar 10 μm. Inset 10 μm B. Polyhedral morphology C. Radial morphology. Actin was labelled with rhodamine phalloidin and Arp2/3 complex with an antibody to the p34-Arc subunit. Expressing cells were identified by labelling with an antibody to myc (not shown).

Since the Arp2/3 complex in filopodia co-localized with lamellipodial markers such as free barbed ends, cortactin and capping protein (data not shown and [17]), we tested whether Arp2/3 complex localisation to filopodia was Rac1 dependent. As previously described [18], expression of the dominant negative form of Cdc42 completely blocked spreading of MEFs (data not shown). Cells expressing dominant negative T17N Rac1 showed a reduction in cell area and complete loss of lamellipodia (data not shown). After 60 minutes of spreading Arp2/3 complex labelling of filopodia in T17N Rac expressing cells was very weak (Figure 8A, arrowheads) despite the fact that the labelling of cytoplasmic actin spots with Arp2/3 complex was normal (Figure 8A, inset box). We concluded that inhibiting lamellipodia with T17N Rac interfered with Arp2/3 complex localisation to filopodia.

Expression of dominant negative T17N Rac1 caused a significant reduction in the number of filopodia compared to untransfected controls (Table 2). Following Rac inhibition, filopodia appeared uniform and straight and clubbed filopodia made up 30% of filopodia (as opposed to 3% without Rac1 inhibition, S. A. Johnston, Ph.D. Thesis) after 60 minutes spreading (Table 3). We concluded that Rac was not essential for the formation of filopodia during spreading but does have a role in their assembly and morphology.

**Table 2:** Number of filopodia per cell in untransfected and dominant negative Rac1 expressing cells spread on fibronection.

	**15 minutes**	**30 minutes**	**60 minutes**
Mock transfected	11.7 (± 2.5)	14.1 (± 1.3)	17.3 (± 3.1)
T17N Rac1	7.7 (± 1.0)	9.4 (± 1.0)	8.3 (± 1.7)

Counts are from 3 independent experiments and are ± standard deviation. Mock transfected, 15 minutes n = 73; 30 minutes n = 50; 60 minutes n = 30. N17 Rac1, 15 minutes n = 47; 30 minutes n = 50; 60 minutes n = 46.

**Table 3:** Incidence of angled, branched and clubbed filopodia.

**T17N Rac1**	**Average number/cell**	**Percentage of total filopodia**
Angled	0	0
Divided or branched	0	0
Clubbed	2.45 (± 0.25)	30

Incidence of angled, branched and clubbed filopodia per cell in dominant negative Rac1 expressing cells spread on fibronection for 60 minutes. Counts are from 27 cells in 3 independent experiments and are ± standard deviation.

Expression of active Rac1 causes constitutive lamellipodia formation (Figure 8). When we spread MEFs expressing constitutively active Rac1 two different morphologies were present: radial (Figure 8C) and polyhedral (Figure 8B). Radial cells were the predominant phenotype with polyhedral cells commonly making up 20–30% of the population at 60 minutes and the polyhedral phenotype was absent in active Rac1 expressing cells prior to trypsin treatment and spreading (data not shown). While radial cells lacked filopodia, polyhedral cells showed a remarkable induction of Arp2/3 complex to their filopodia with the vast majority showing labelling (Figure 8B).

## Discussion

Our data suggest that Arp2/3 complex is localised to both the filopodia and lamellipodia of spreading fibroblasts. While Arp2/3 complex is typically thought of as a lamellipodial protein, it has been observed in filopodia of *Dictyostelium *and *Acanthamoeba *[11,19] and can crosslink actin filaments in addition to the much more well-characterised actin nucleation properties [20]. There have been mixed reports about whether Arp2/3 complex localizes to mammalian cell filopodia. In B16F1 melanoma cells, Arp2/3 complex was reported to be excluded from filopodia [4], while a previous study stated that Arp2/3 complex was only excluded from most filopodia in fibroblasts [21]. Although Arp2/3 complex has been observed in filopodia, its function here has not been well defined, perhaps because it is a relatively minor component of filopodia and very prominent in lamellipodia. However, the presence of Arp2/3 complex in filopodia may have implications for our understanding of their dynamics.

Just as lamellipodial actin feeds into other actin structures such as cell junctions [22], the actin network generated by Arp2/3 complex in filopodia may provide filaments of different orientations for filopodia dynamics or for assembly of other structures [23]. We have found that Arp2/3 complex is present in angled and clubbed filopodia, both situations where non-linear filaments could be present (unpublished data, S. A. Johnston, Ph.D. Thesis). Of course careful electron microscope studies would be needed to determine if branched networks could be formed in filopodia, but our preliminary results using critical point drying and platinum replica methods [24] show that lamellipodial networks do sometimes protrude from filopodia of MEF (unpublished data, S. A. Johnston, Ph.D. thesis). Importantly, the existence of stable branched networks even in lamellipodia has recently been called into question by one group, who propose that critical point drying methods of preparation for electron microscopy may promote branching of filaments[25]. Although lamellipodial protrusions from filopodia may have been observed previously, this is not widely discussed in the literature. Short actin filaments have been observed to occur in the tips of *Dictyostelium *filopodia [23] and to be associated with the elongating "tip complex" of these filopodia. We did see Arp2/3 complex in the tips of clubbed filopodia, which may have correlated with the tip complex.

The formation of new adhesions is occurring during spreading and was an obvious possibility to explain the presence of Arp2/3 complex in filopodia. DeMali et al. showed [12] that vinculin transiently associated with Arp2/3 complex during spreading and that this association might promote actin assembly at or near new adhesions. Most of the Arp2/3 complex we saw in filopodia did not co-localise with vinculin or with adhesion sites. However, we did frequently see Arp2/3 complex co-localisation with vinculin at bends and angled regions of filopodia (unpublished data, S. A. Johnston, Ph.D. Thesis). It is still possible that Arp2/3 complex is transiently recruited to new adhesions, as is also suggested by recent work demonstrating a connection with focal adhesion kinase [26], but vinculin binding does not appear to be required for the recruitment of Arp2/3 complex to filopodia, nor does adhesion to substratum.

Arp2/3 complex in filopodia could be as a precursor to the lamellipodial expansion that is often seen in spreading cells, and which we observed with MEFs (see Additional file 1). In agreement with a model where Arp2/3 complex is recruited to nascent filopodia in order to promote spreading of cells and progression to lamellipodia, we found that Rac inhibition dramatically decreased localisation of Arp2/3 complex to filopodia. Arp2/3 complex in filopodia was distinguishable from localisation to persistent lamellipodia that have been shown to be sensitive to nocodazole treatment [27] and dependent on adhesion [28]. The localisation of Arp2/3 complex in filopodia is upstream of adhesion formation and receptor engagement although whether Arp2/3 complex is actively producing an actin network in this situation is unclear. However, we never observed a single spot of Arp2/3 complex in a filopodium progress to become a lamellipodial protrusion, so either this is a low-frequency event or an additional event needs to take place, such as coalescence of several Arp2/3-rich areas within a filopod for lamellipodial progression to occur.

Our initial results with fluorescently labelled actin monomers suggest that there are free barbed ends near the sites of Arp2/3 complex enrichment in filopodia. Recently it has been shown that Rac is crucial is for the stabilization of protrusion [29] and perhaps the distinction between the characteristics of Arp2/3 complex in filopodia is due to its involvement in this stabilization.

Svitkina and colleagues have described a role for the filopodial protein mDia2 in lamellipodia assembly and a molecular connection between the Scar/WAVE complex protein Abi1 and mDia2 [30]. Thus, they also propose crosstalk between filopodia and lamellipodia. While it is clear that full activity of Arp2/3 complex is not necessary for the generation of filopodia in all cells and conditions [5], these structures can be interdependent [3]. In a 3-dimensional network, crawling cells likely have to change between filopodial and lamellipodial protrusions as well as navigating turns and bends more than on a glass coverslip, so it will be interesting to determine whether filopodia made in 3D also have lamellipodial elements.

## Conclusion

Our results suggest that lamellipodial networks may exist in filopodia and that these may be important in the dynamics of filopodia or perhaps in transitions between lamellipodial and filopodial modes of protrusion. Our results also suggest that Arp2/3 complex networks do not necessarily only contribute to lamellipodia or remain branched in cells, in agreement with other recent work [31,32].

## Methods

### Cell culture and transfection

Mouse embryonic fibroblasts (MEF) where derived from E9 embryos and immortalised in culture [33]. Vinculin null MEF were a gift from Eileen Adamson (Irvine, USA). Vin -/- MEFs were derived from E10.5 embryos and immortalised in culture [13]. J774.A1 macrophages (a gift of Juergen Wehland, Braunschweig, DE) were plated on 13 mm cover slips in Dulbeccos's modified Eagles medium (DMEM) with 10% foetal calf serum for 2 hours at 37°C in a humidified atmosphere of 5% CO_2 _and 95% air, fixed and processed for immunofluorescence as described below. All cells were grown in Dulbeccos's modified Eagles medium (DMEM; Invitrogen, Paisley, UK) with 10% foetal calf serum, glutamine, 50 U/ml penicillin and 50 μg/ml streptomycin at 37°C in a humidified atmosphere of 5% CO_2 _and 95% air. For transfection, cells were plated at 2 × 10^4 ^cells/well in 4-well dishes (Fisher, Loughborough, UK), after 24 hrs a mixture of DNA and Lipofectamine 2000 (Invitrogen, Paisley, UK) 4:1 (w/v) was added in antibiotic free media. Cells were used in experiments 24 hrs after transfection.

### DNA Reagents

Expression constructs for RFP non-muscle β-actin was a gift from Maddie Parsons (London, UK), Capping protein CPβ2-GFP was a gift from John Cooper (St. Loius, USA [17]), GFP-fascin was a gift of Josephine Adams (Cleveland, USA), Rho GTPase contructs were gifts from Alan Hall (New York, USA) and p21-arcGFP was a gift from Jurgen Wehland (Braunschweig, DE).

### Cell Spreading Assay

Coverslips were acid washed and coated with 10 μg/ml fibronectin from human plasma (F-2006 Sigma, Poole, UK) in PBS for 1 hr at RT. After coating, coverslips were washed three times in PBS and blocked with 10 mg/ml denatured BSA (fatty acid free A8806; Sigma, Poole, UK) for 1 hr RT. Cells are unable to spread or lay down ECM components on glass blocked with BSA in this way [15]. Sub-confluent MEFs were trypsinised, suspended in DMEM supplemented with 0.5 mg/ml Trypsin inhibitor from soy bean (Sigma, Poole, UK) and diluted to 4 × 10^4 ^cells/ml in DMEM. For live imaging, cells were suspended in Tyrodes buffer [34] instead of DMEM.

### BSA blocking of cell spreading

MEFs were plated at 2 × 10^4 ^cells/well in DMEM on 13 mm acid washed glass cover slips and were incubated for 5 minutes at 37°C in a humidified atmosphere of 5% CO_2_. After 5 minutes, medium was replaced with DMEM containing 10 mg/ml denatured BSA (fatty acid free A8806; Sigma, Poole, UK) and cells incubated until 60 minutes after plating at 37°C in a humidified atmosphere of 5% CO_2_.

### Immunocytochemistry

Primary antibodies used are: polyclonal anti-p34-arc (a member of the Arp2/3 complex; Rabbit; Upstate, NY, US; unless otherwise indicated this antibody is used to localise Arp2/3 complex), monoclonal anti-Arp2 (a member of the Arp2/3 complex; Mouse; Sigma, Poole, UK), monoclonal anti-myc, clone 9E10 (mouse; Sigma, Poole, UK), monoclonal anti-vinculin clone, hVin-1 (mouse; Sigma, Poole, UK), monoclonal anti-cortactin, clone 4F11 (mouse; Upstate, NY, US) and monoclonal paxillin, clone 349 (mouse; BD Biosciences, San Jose, US). Fluorescently labelled phalloidins used are Alexa350 (Invitrogen, Paisley, UK) and TRITC (Sigma, Poole, UK). Cells were fixed in 4% formaldehyde in PBS for ten minutes, washed six times in PBS and quenched in 50 mM NH_4_Cl for ten minutes. Cells were washed three times in PBS, made permeable in 0.1% Triton X-100 PBS for 4 minutes and transferred without washing into primary antibody for twenty minutes. After primary antibody labelling cells were washed three times in PBS and transferred to secondary antibodies for twenty minutes. When labelling with a biotin-conjugated antibody, Cascade Blue labelled avidin (Invitrogen, Paisley, UK) was added as a third labelling step. Phalloidin was added with the final labelling step. After labelling cells were washed three times in PBS, three times in dH_2_O and mounted in Mowiol (Calbiochem, Nottingham, UK) with p-phenylene diamine (Sigma, Poole, UK) as an antifade agent.

### Fluorescent Microscopy of Fixed Cells and Image Processing

A Zeiss Axioskop2 microscope with a 63× 1.4NA Plan Apochromat lens was used for fluorescent microscopy of fixed cells. Images were captured using a Qimaging 12-bit QICAM with a 0.63× camera lens and Openlab software (Improvision, Coventry, UK) and saved as 12-bit tif files. Images were processed for publication in Adobe Photoshop (Adobe). The image histogram was adjusted to make full use of the available greys, without adjusting gamma, converted to 8-bit and where necessary merged with corresponding images, taken with different fluorescent filters, to make an 8-bit/channel RGB file. Regions of interest where selected from merged images and scale bars were added. Where image size was adjusted for making figures this was done in Photoshop without resampling of the image.

### Time-Lapse Microscopy of Live Cells

Cells where plated onto a 25 mm cover slip in a cell chamber from Medical Systems Corp. (Greenville, NY), the chamber was filled with media or buffer and sealed with a second 25 mm coverslip. A Zeiss Axiovert100 microscope was kept at 37°C in a custom built incubation chamber (Solent Scientific). Images were captured using a Hammatsu Orca C4745-12NRB with a 0.5× camera lens and Openlab software (Improvision, Coventry, UK).

For correlations between DIC microscopy and fixed fluorescent microscopy, cells were spread on cover slips that had photo-etched numbered squares (Electron Microscopy Sciences, Washington, USA). Etched cover slips were coated with fibronectin as described above. Cells were plated onto cover slips and imaged for the required time, the numbered square imaged was noted for finding the same field after fixation. Cells were fixed with 4% formaldehyde while on the microscope stage. After fixation cells were processed for immunofluorescence as described above.

### Measurements and counts of cells

Image stacks used for measurement and counts of cells were 12-bit images normalised with 0.5% saturated pixels using the stack histogram in ImageJ (NIH, Bethesda, USA). All counts were done on the same CRT screen with identical brightness and contrast. We counted the number of filopodia and retraction fibres at 15, 30 and 60 minutes after plating from DIC microscopy time lapse sequences. Filopodia were defined as thin projections that grew from the cell edge and whose tips moved relative the cell body [10]. Retraction fibres were defined as thin projections that were left behind after the retraction of a previously spread area, their tips were static compared to the cell body. When counting the number of filopodia of fixed cells filopodia were defined as bundled actin structures that extended beyond the cell edge. Filopodia and retraction fibres cannot be distinguished in this way.

Angled filopodia had a greater than 45 degree bend at a single inflection point. Divided filopodia were a single filopodium that split into two, either as filopodium with a side-branch or an equal division into two filopodia. Clubbed filopodia had an increase in F-actin at their tips (most filopodia when labelled with phalloidin have decreased F-actin tip labelling).

### Drug treatments

For microtubule depolymerization experiments, trypsinised cells were diluted to 4 × 10^4 ^cells/ml in DMEM with 2.5 μg/ml nocodazole. Cells were incubated for 5 minutes in suspension with nocodazole and then plated onto fibronectin cover slips, fixed and processed for immunofluorescence as described above.

### Labelling of free barbed ends with Cy3 actin

Cy3 actin was prepared as previously described [35]. Spreading cells were permeabilised and labelled with Cy3 actin for 3 minutes in 20 mM Hepes, pH 7.5, 138 mM KCl, 4 mM MgCl_2_, 3 mM EGTA, 0.2 mg/ml Saponin, 1 mM ATP, 1% BSA and 0.45 μm Cy3 Actin at room temperature. After permeabilisation and labelling cells were fixed with 4% formaldehyde for 10 minutes. After fixation cells were processed for immunofluorescence as described above.

### Stamp Preparation for microcontact printing

Microcontact printing is a 4-step process: Formation of silicon masters, casting of stamps from master, inking of stamp, printing of pattern. Two different polydimethylsiloxanes (PDMS) stamp configurations were used for the microcontact printing experiments. 10 μm wide strips separated by 10 μm gap and 5 μm wide strips separated by 5 μm gaps. Standard photolithography techniques were used to produce the micropatterned silicon masters. PDMS stamps were fabricated by casting a 10:1 (v:v) mixture of PDMS-Sylgard 184 Silicone Elastomer Base (Dow Corning) and 184 Sylgard Curing Agent (Dow Corning) onto the micropatterned silicon masters. After allowing the PDMS stamps to degas at ambient conditions for 1 h, the stamps were cured for 2 h at 60°C. The solidified PDMS stamps were then carefully peeled from the masters. In order to localise micropatterns fibronectin was labelled with the fluorophore Cy3. Cy3 as a bisfunctional NHS-ester (Amersham, Buckingham, UK) was coupled to fibronectin in 0.1 M sodium carbonate buffer (pH 9.3) for 30 minutes at room temperature. Cy3 fibronectin was dialysed against 1 L PBS for 24 hours with two changes of buffer. To produce pattern unlabelled fibronectin was doped with Cy3 fibronectin in a 4:1 ratio. Stamps where inked for between 5 and 40 minutes, washed and placed in contact with glass cover slip. To block uncoated areas of the pattern the surfaces were coated with heat denatured bovine serum albumin (BSA) for 1 hour [15].

## Abbreviations

Arp2/3 complex: actin related protein 2/3 complex; GTPase: guanidine triphosphatase; Arp2: actin related protein 2; Arp3: actin related protein 3; WASP: Wiskott-Aldrich syndrome protein; VASP: vasodilator-stimulated phosphoprotein; Scar/WAVE complex: suppressor of cyclic adeonosine monophosphate receptor/WASP-family verprolin homologous protein complex; MEFs: mouse embryonic fibroblasts; DIC: differential interference contrast; GFP: green fluorescent protein; RFP: red fluorescent protein; BSA: bovine serum albumin; mDia2: mammalian Diaphanous-related formin 2.

## Authors' contributions

SAJ did all of the experimental work. Together, LMM and SAJ conceived the study, designed experiments and wrote the manuscript. JPB, CLY and PMM designed and produced the microcontact surfaces. PMM also contributed to the experimental design, editing and ideas for the manuscript.

## Supplementary Material

Additional file 1 **Movie of morphology of mouse embryonic fibroblast spreading on fibronectin**. MEFs spread through cycles of filopodia and lamellipodia production making them excellent for the study of both these structures. MEFs in suspension were plated onto fibronectin coated coverslips and a field of view was selected at random. Lines that can be seen on the coverslip are numbered areas for locating cells for correlative fluorescence microscopy. Cells spread identically on marked coverslips compared to unmarked coverslips in all experiments. Frames were captured every 10 seconds. This cell is representative of 30 cells from 4 independent experiments. Movie is displayed at 5 frames per second. Click here for file

Additional file 2 **Morphology of mouse embryonic fibroblast spreading on fibronectin**. A. Mouse embryonic fibroblasts spread with cycles of filopodia and then lamellipodia. MEFs were plated on fibronectin and a timelapse sequence of DIC images taken up to 60 minutes after plating. Images shown here are frames taken at times indicated after plating. Scale bar 10 μm. B. Total spread area was measured from cells spread for times indicated and then fixed. Data are from three experiments, 15' n = 73, 30' n = 66 and 60' n = 66. Click here for file

Additional file 3 **Double antibody labelling Arp2/3 complex in filopodia**. Antibodies to the Arp2/3 complex members p34-arc and arp2 both localise Arp2/3 complex to filopodia. Cells were spread on fibronectin for 60 minutes and co-labelled with a rabbit polyclonal antibody to p34-arc and a mouse monoclonal antibody to Arp2. B and D (scale bar 2 μm) are enlargements of boxed area in A and C (scale bar 10 μm) respectively. Click here for file

Additional file 4 **Example of protruding filopodia containing Arp2/3 complex**. This is an example of a protruding filopodium that contains Arp2/3 complex (still pictures are shown in Figure 2). Movie 3 is a cropped region of interest of a MEF that was spread for 60 minutes on a fibronectin coated coverslip. The tip of the filopodium extends towards the bottom left hand corner up until fixation for correlative fluorescent microscopy. Frames were captured every 10 seconds for 60 minutes. Movie contains the last 3 minutes of this timelapse. Still pictures are shown in Figure 2. Movie is displayed at 5 frames per second. Click here for file

Additional file 5 **Movie of localisation of Arp2/3 complex to filopodia**. Arp2/3 complex localises to filopodia of live cells at multiple sites. A GFP tagged p21-arc subunit of Arp2/3 complex and mRFP tagged actin were transiently transfected into MEFs. After spreading on fibronectin for 60 minutes low expressing cells were identified and timelapse sequences taken. Movie 3 is representative of 11 cells in 6 independent experiments. Frames were captured every 10 seconds and movie is displayed at 5 frames per second. Click here for file

Additional file 6 **Dynamics of Arp2/3 complex in filopodia**. Arp2/3 complex patches in filopodia are highly dynamic. Movie 4 is a cropped region of interest from Movie 3. Arrows were added in Openlab 4 (Improvision, UK). Movie is displayed at 5 frames per second. Click here for file

Additional file 7 **Arp2/3 localises to dynamic and static filopodia**. A) Arp2/3 complex containing filopodium from a cell that is actively protruding. A patch of Arp2/3 complex appears in a filopodium before being overtaken by the extending lamellipodium. Images are taken from timelapse movie frames captured every 10 seconds. Scale bar 5 μm. B) Arp2/3 complex containing filopodium from a cell that is not actively protruding. The Arp2/3 complex in this situation is much less dynamic than that seen in additional file 7. This suggests a correlation between the protrusive activity at the site of an Arp2/3 complex containing filopodium and the dynamics of Arp2/3 complex in that filopodium. Images are taken from timelapse movie frames captured every 10 seconds. Scale bar 5 μm. Click here for file

Additional file 8 **Blocking new adhesion formation of spreading mouse embryonic fibroblasts**. Lamellipodia but not filopodia are dependent on formation of new adhesions. After 5 minutes of attachment to a glass cover slip, the surface was blocked with denatured BSA and cells were allowed to spread for a further 55 minutes before fixation. Actin was labelled with rhodamine phalloidin and vinculin and Arp2/3 complex with antibodies. Scale bar 10 μm. Click here for file

Additional file 9 **Formation of lamellipodia and filopodia on micro-patterned surfaces**. Lamellipodia but not filopodia are dependent on an adhesive surface. A. Cells were spread on 5 μm fibronectin and BSA stripes for 60 minutes. Scale bar 10 μm B. Cells were spread on 10 μm fibronectin and BSA stripes for 60 minutes. Scale bar 10 μm. Hollow arrows indicate filopodia. Solid arrows indicate lamellipodia/ruffles. Actin was labelled with Alexa 350 phalloidin. Scale bar 10 μm. Click here for file
