# Supplementary figures and images for: Arp2/3 complex activity in filopodia of spreading cells
Source: BMC Cell Biol. 2008 Dec 9;9:65. doi: 10.1186/1471-2121-9-65 (PMC2639383; doi:10.1186/1471-2121-9-65)

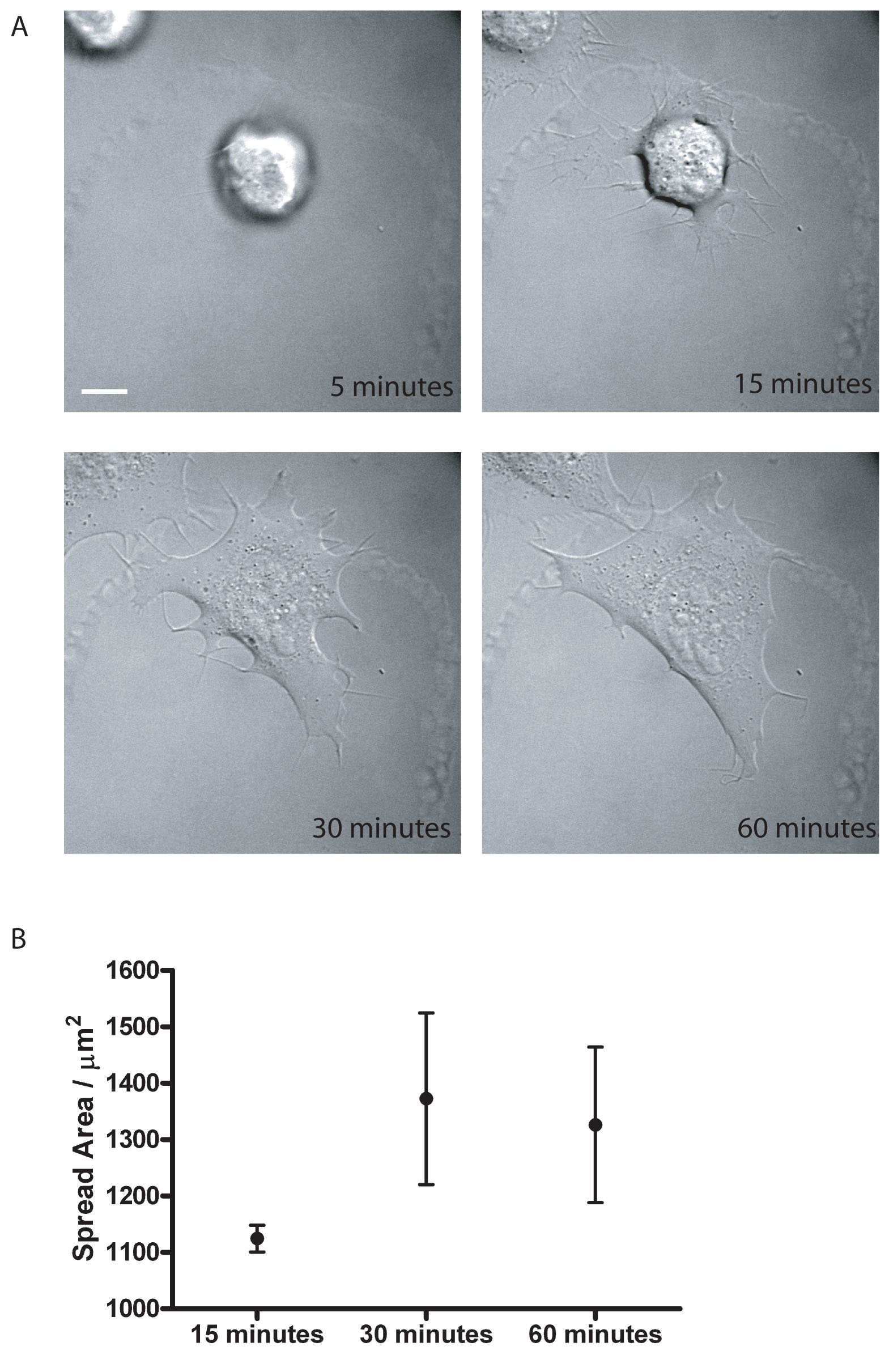

Supplement: Additional file 2 — Morphology of mouse embryonic fibroblast spreading on fibronectin. A. Mouse embryonic fibroblasts spread with cycles of filopodia and then lamellipodia. MEFs were plated on fibronectin and a timelapse sequence of DIC images taken up to 60 minutes after plating. Images shown here are frames taken at times indicated after plating. Scale bar 10 μm. B. Total spread area was measured from cells spread for times indicated and then fixed. Data are from three experiments, 15' n = 73, 30' n = 66 and 60' n = 66. [file 1471-2121-9-65-S2.jpeg]

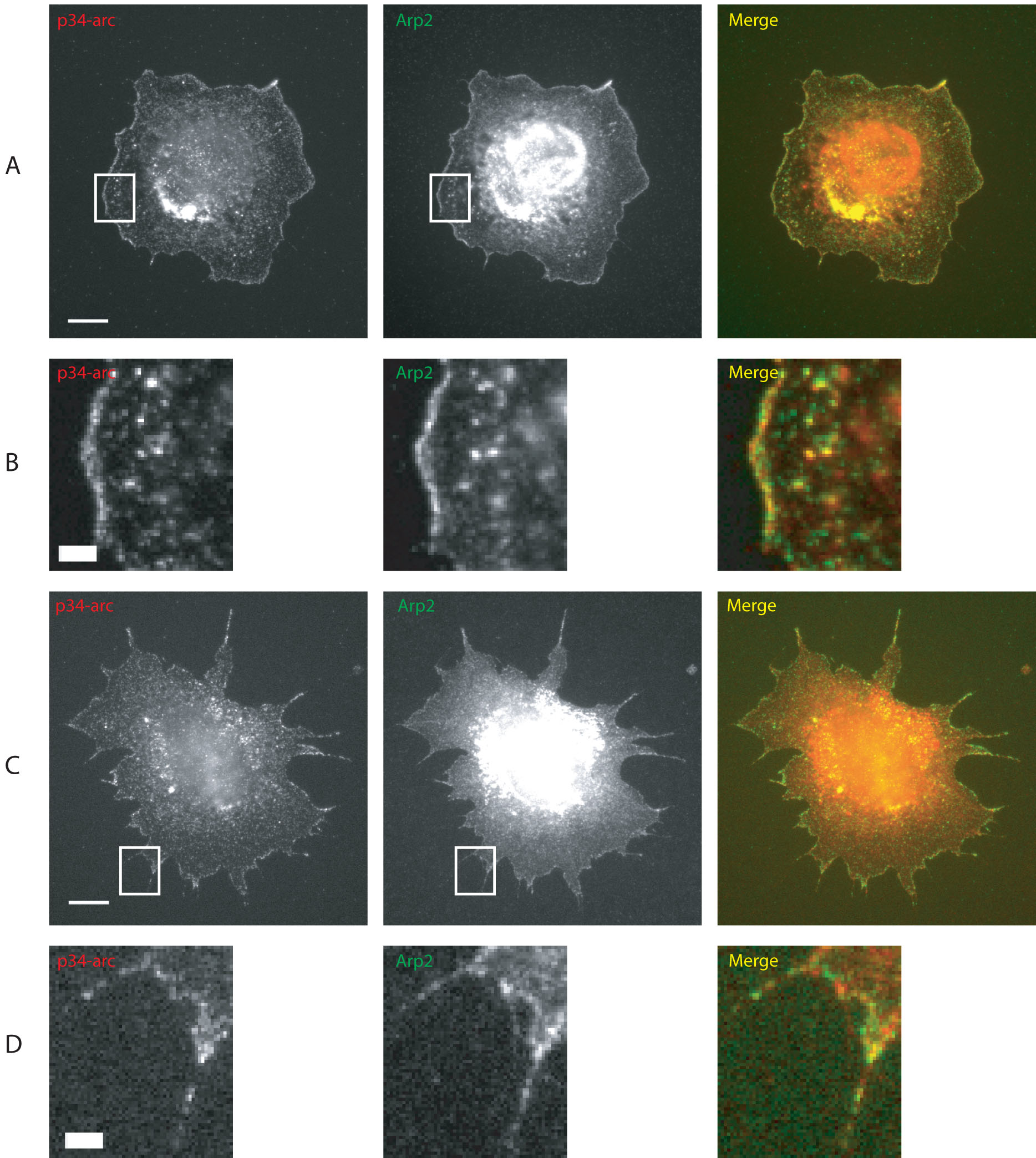

Supplement: Additional file 3 — Double antibody labelling Arp2/3 complex in filopodia. Antibodies to the Arp2/3 complex members p34-arc and arp2 both localise Arp2/3 complex to filopodia. Cells were spread on fibronectin for 60 minutes and co-labelled with a rabbit polyclonal antibody to p34-arc and a mouse monoclonal antibody to Arp2. B and D (scale bar 2 μm) are enlargements of boxed area in A and C (scale bar 10 μm) respectively. [file 1471-2121-9-65-S3.jpeg]

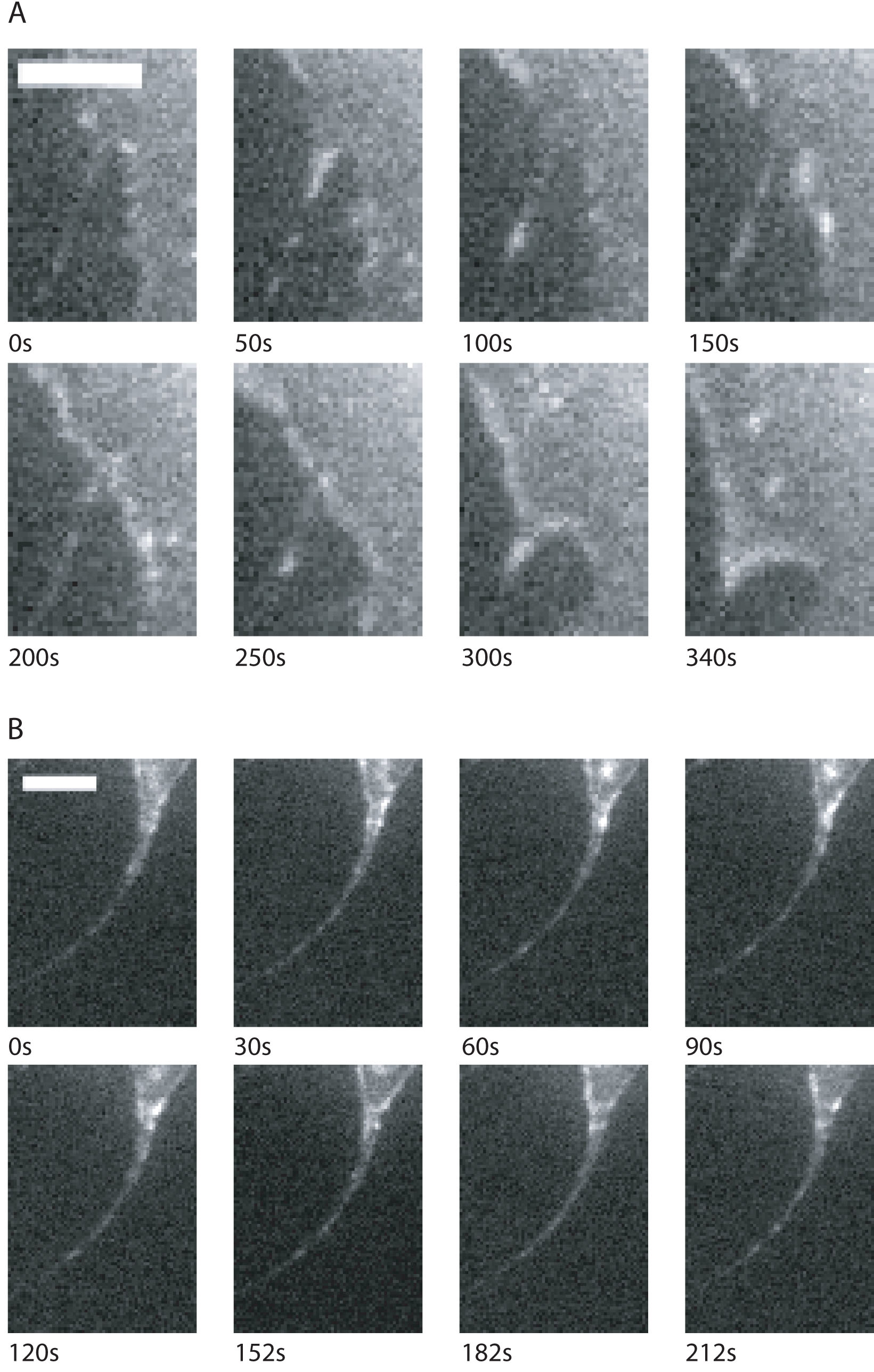

Supplement: Additional file 7 — Arp2/3 localises to dynamic and static filopodia. A) Arp2/3 complex containing filopodium from a cell that is actively protruding. A patch of Arp2/3 complex appears in a filopodium before being overtaken by the extending lamellipodium. Images are taken from timelapse movie frames captured every 10 seconds. Scale bar 5 μm. B) Arp2/3 complex containing filopodium from a cell that is not actively protruding. The Arp2/3 complex in this situation is much less dynamic than that seen in additional file 7. This suggests a correlation between the protrusive activity at the site of an Arp2/3 complex containing filopodium and the dynamics of Arp2/3 complex in that filopodium. Images are taken from timelapse movie frames captured every 10 seconds. Scale bar 5 μm. [file 1471-2121-9-65-S7.jpeg]

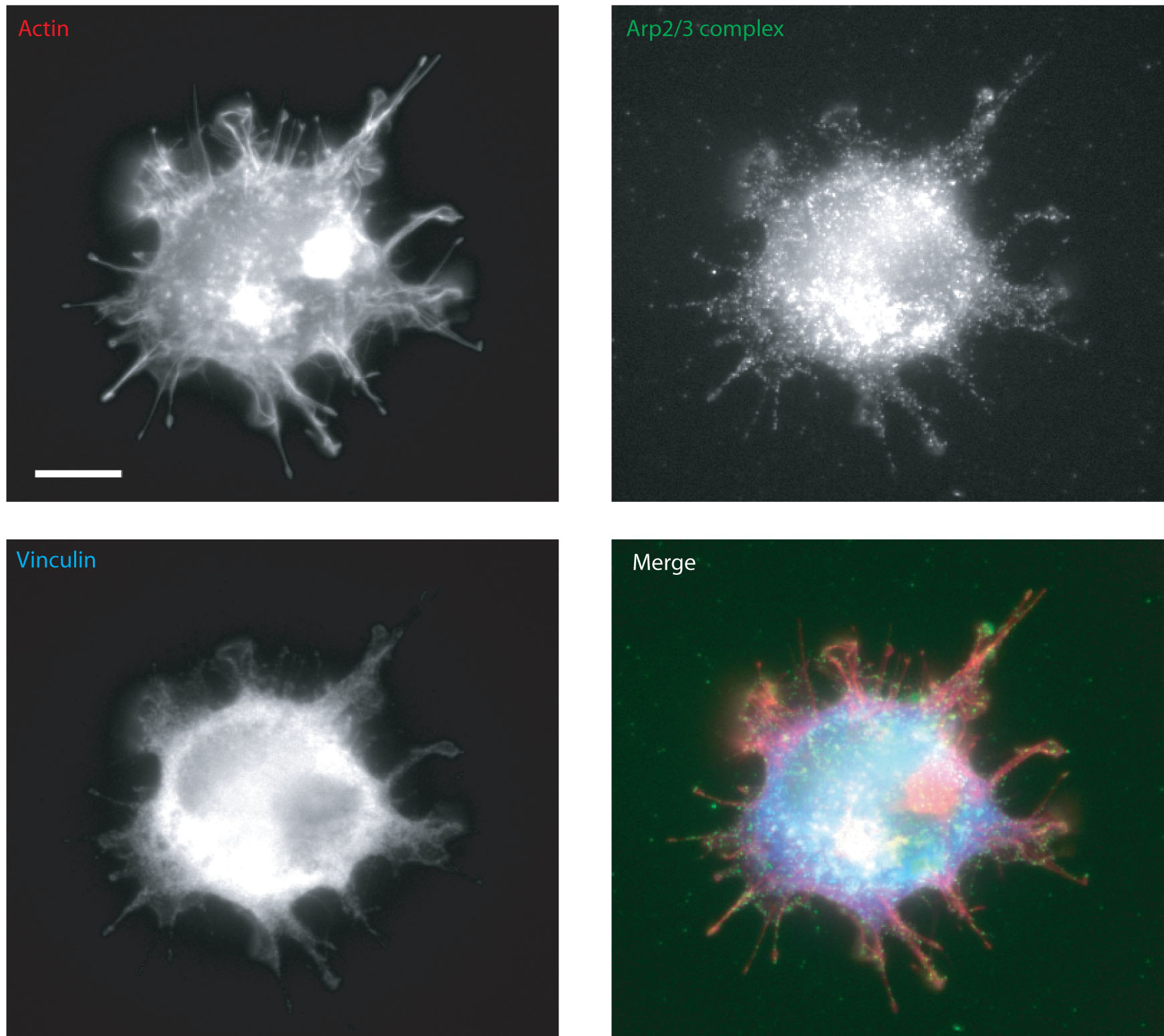

Supplement: Additional file 8 — Blocking new adhesion formation of spreading mouse embryonic fibroblasts. Lamellipodia but not filopodia are dependent on formation of new adhesions. After 5 minutes of attachment to a glass cover slip, the surface was blocked with denatured BSA and cells were allowed to spread for a further 55 minutes before fixation. Actin was labelled with rhodamine phalloidin and vinculin and Arp2/3 complex with antibodies. Scale bar 10 μm. [file 1471-2121-9-65-S8.jpeg]

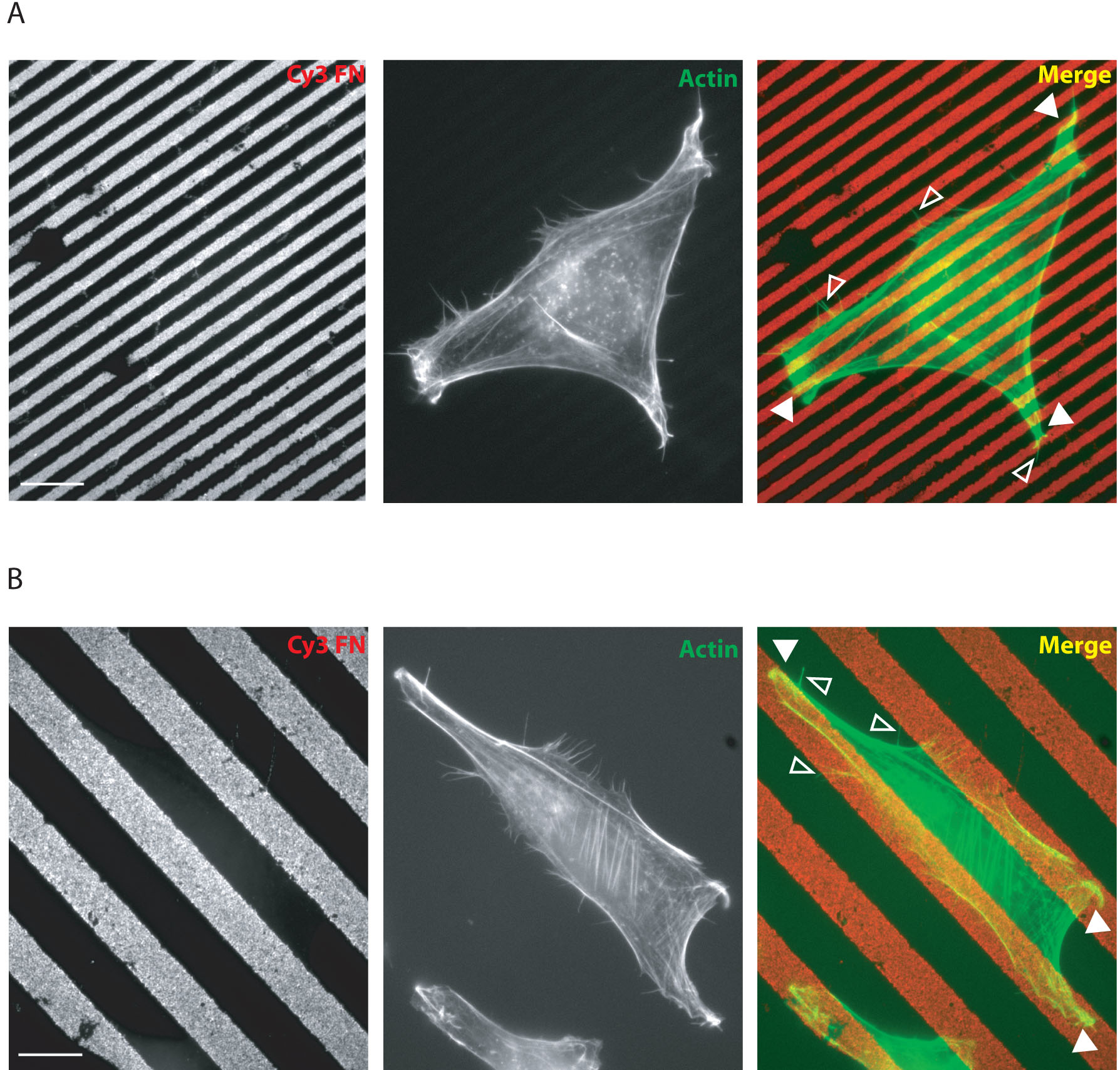

Supplement: Additional file 9 — Formation of lamellipodia and filopodia on micro-patterned surfaces. Lamellipodia but not filopodia are dependent on an adhesive surface. A. Cells were spread on 5 μm fibronectin and BSA stripes for 60 minutes. Scale bar 10 μm B. Cells were spread on 10 μm fibronectin and BSA stripes for 60 minutes. Scale bar 10 μm. Hollow arrows indicate filopodia. Solid arrows indicate lamellipodia/ruffles. Actin was labelled with Alexa 350 phalloidin. Scale bar 10 μm. [file 1471-2121-9-65-S9.jpeg]
